# The efficacy and safety of vonoprazan in quadruple therapy for *Helicobacter pylori* eradication: a comparative study

**DOI:** 10.1093/gastro/goae036

**Published:** 2024-04-15

**Authors:** Xiaoduan Zhuang, Huiyue Jiang, Dandan Jin, Meiling Sun, Zhenwu Wang, Xinying Wang

**Affiliations:** Department of Gastroenterology, Zhujiang Hospital, Southern Medical University, Guangzhou, Guangdong, P. R. China; Department of Gastroenterology, Zhujiang Hospital, Southern Medical University, Guangzhou, Guangdong, P. R. China; Department of Gastroenterology, Zhujiang Hospital, Southern Medical University, Guangzhou, Guangdong, P. R. China; Department of Gastroenterology, Zhujiang Hospital, Southern Medical University, Guangzhou, Guangdong, P. R. China; Department of Gastroenterology, Zhujiang Hospital, Southern Medical University, Guangzhou, Guangdong, P. R. China; Department of Gastroenterology, Zhujiang Hospital, Southern Medical University, Guangzhou, Guangdong, P. R. China

**Keywords:** Vonoprazan, proton pump inhibitors, *Helicobacter pylori* eradication, Quadruple therapy

## Abstract

**Background:**

The efficacy and optimal dose of the new acid-suppressant vonoprazan (VPZ) for quadruple therapy remain uncertain. This study aimed to compare the efficacy and safety of 20 mg VPZ daily (VOD) and 20 mg VPZ twice daily (VTD) with a proton pump inhibitor (PPI) twice daily in quadruple therapy.

**Methods:**

We retrospectively analyzed the data of 954 patients treated with quadruple therapy to eradicate *Helicobacter pylori*. Eradication rates and adverse events were compared between the VOD and VTD groups, and between the VOD and PPI groups. Multivariate analysis was conducted to identify the predictors of eradication failure.

**Results:**

Eradication was successful in 875 (91.7%) of the 954 patients. The total, initial, and rescue eradication rates in the VOD group were 92.1%, 93.3%, and 77.8%, respectively. In both the crude and multivariate analyses, the VOD group showed eradication rates comparable to those of the VTD and PPI groups (all *P *>* *0.05). Age > 60 years (odds ratio [OR] = 2.165, *P *=* *0.012) and use of rescue therapy (OR = 3.496, *P *<* *0.001) were independent risk factors for eradication failure, whereas VPZ at a low dosing frequency of 20 mg daily was not. A total of 787 patients (82.5%) were followed up (mean follow-up time, 6.7 ± 2.0 months). Compared with the VOD group, the VTD group was more likely to experience adverse events (OR = 2.073, *P *=* *0.035).

**Conclusion:**

VPZ at a low dose of 20 mg daily is an effective and safe component of the quadruple therapy for *H.pylori* eradication.

## Introduction

*Helicobacter pylori* (*H.pylori*) has been reported to infect approximately 50% of the worldwide population [1, 2]. Its presence is an identified risk factor for a variety of gastrointestinal and extra-gastric diseases, including chronic gastritis, peptic ulcers, gastric carcinoma, and iron-deficiency anemia. Quadruple regimens are recommended as first-line treatment in areas with high *H.pylori* clarithromycin resistance [3–6]. Bismuth-containing quadruple therapy is recommended as an empirical therapy to eradicate *H.pylori* infection in China [6]. An empiric eradication regimen is considered acceptable if it reliably achieves ≥ 90% eradication rates [7]; however, *H.pylori* eradication rates of the bismuth-containing quadruple therapy regimen are not always satisfactory in different areas [8, 9]. To date, various attempts have been made to overcome the declining eradication rates, including the use of antibiotics with low antimicrobial resistance, increasing drug dosages, and cytochrome CYP2C19 genotyping. Among these, a stronger acid inhibitor that maintains adequate acid inhibition may be a breakthrough in maintaining and even improving *H.pylori* eradication rates. Maintenance of a high gastric pH not only improves the gastric bioavailability of antibiotics but also makes *H.pylori* more susceptible to antibiotics [10, 11].

Vonoprazan (VPZ), first introduced in Japan in 2015, is a novel potassium-competitive acid blocker. Previous studies have shown that VPZ-based regimens can achieve significantly higher *H.pylori* eradication rates and are promising alternatives to the currently available proton pump inhibitors (PPI)-containing regimens [12–15]. Notably, the above findings were predominantly constrained to triple or high-dose dual regimens and the efficacy of VPZ-containing quadruple therapies remains unclear. In addition, there is no consensus regarding the dosing frequency of VPZ in the quadruple regimen. In previous studies, 20 mg VPZ was administered twice daily to eradicate *H.pylori* infection in triple or high-dose dual regimens [14–16]. However, it remains unclear whether VPZ 20 mg daily has sufficient acid-inhibiting effects to assist *H.pylori* eradication in empirical quadruple therapies.

Therefore, our study aimed to assess the efficacy and safety of the VPZ-containing quadruple regimen and to determine whether a low dosing frequency of VPZ 20 mg daily as part of the quadruple regimen was as effective as the approved PPI-containing quadruple regimen.

## Materials and methods

### Study cohort and approval

This retrospective study was based on the medical records of patients diagnosed with *H.pylori* infection at Zhujiang Hospital, affiliated with Southern Medical University (Guangdong, P. R. China), between January 2022 and December 2022. The initial inclusion criteria were as follows: (i) patients with current *H.pylori* infection detected using the ^13^C-urea breath test (UBT) and/or rapid urease test; (ii) patients who received the 14-day empirical quadruple therapy (potassium-competitive acid blocker/PPI + bismuth potassium citrate + amoxicillin + clarithromycin) for *H.pylori* infection, and (iii) patients who underwent a ^13^C-UBT assessment to determine the eradication of *H.pylori* infection by 4–12 weeks after treatment completion. The exclusion criteria were as follows: (i) age <18 years old; (ii) a history of distal gastrectomy; (iii) concurrent use of probiotics, histamine H2 antagonists, or Chinese medicine during the period of *H.pylori* eradication; and (iv) incomplete medical records. For rescue therapy, patients with unclear treatment records were considered to have incomplete medical records. This study was approved by the Clinical Research Ethics Committee of Zhujiang Hospital (approval No. 2023-KY-119) and conducted in accordance with the Ethical Principles for Medical Research Involving Human Subjects, as defined in the Helsinki Declaration.

### Diagnosis and eradication of *Helicobacter pylori*

^13^C-UBT and/or a rapid urease test were used to detect the current infection of *H.pylori* prior to treatment. To avoid ^13^C-UBT false negative results, no acid-suppressing drugs or antibiotics were administered at least two weeks before the test. In the ^13^C-UBT test, a delta over baseline value of 4% or higher was defined as the current infection of *H.pylori*. The patients were treated with the 14-day bismuth-containing quadruple therapy. Except for VPZ, the dosages of other drugs were in accordance with Chinese guidelines: PPI (standard dose twice daily), potassium bismuth citrate (220 mg twice daily), penicillin (1.0 g twice daily), and clarithromycin (500 mg twice daily) [6]. An acid-suppressing drug, PPI or VPZ, was selected as a component of the quadruple therapy. Patients who received VPZ were administered 20 mg VPZ either once or twice daily during the quadruple therapy treatment period, as determined by the physician in charge. According to the different doses of acid-suppression drugs, the included patients were classified into the low dosing frequency (20 mg daily) of VPZ (VOD group), high dosing frequency (20 mg twice/day) of VPZ (VTD group), and standard-dose PPI twice daily (PPI group).

### Outcome measurements

The primary outcome was the total eradication rate of *H.pylori*. Successful eradication was confirmed by ^13^C-UBT 4–12 weeks after the end of quadruple therapy. A delta over baseline value of less than 4% was considered successful eradication.

The secondary outcomes included eradication rates in subgroup analyses (stratified by eradication attempts, acid suppressors, and age), potential risk factors for eradication failure, and adverse events (AEs) during eradication. AEs were defined as undesirable clinical symptoms after the initiation of treatment, which were assessed via face-to-face consults or telephone calls. For patients with overlapping symptoms before and after treatment, we excluded those that overlapped with pre-treatment symptoms from the analysis of AEs. The observed AEs were classified as gastrointestinal or extra-gastrointestinal, including abdominal pain, abdominal distention, constipation, diarrhea or soft stool, dyspepsia, reflux or heartburn, nausea, vomiting, taste disturbance, skin rash, headache, dizziness and anorexia.

### Statistical analysis

Categorical variables and continuous variables were expressed as numbers and percentages, and mean ± standard deviation, respectively. Comparisons of eradication rates and AEs between the VPZ daily (VOD) and VPZ twice daily (VTD) groups or between the VOD and PPI groups were performed using the *t*-test, chi-square test, or Fisher’s exact test. Crude and adjusted logistic regressions were used to calculate the odds ratios (OR) and 95% confidence intervals (CI) for the association between *H.pylori* eradication failure and acid suppression drugs. Adjustments were first made for age and sex (Model 1). Additional adjustments were made for residence, smoking status, diabetes history and eradication attempts (Model 2). All statistical analyses were performed using IBM SPSS Statistics for Windows, version 22.0 (IBM Corporation, Armonk, NY, USA). A two-sided *P *<* *0.05 was considered statistically significant.

## Results

### Patient characteristics

**Figure 1.**
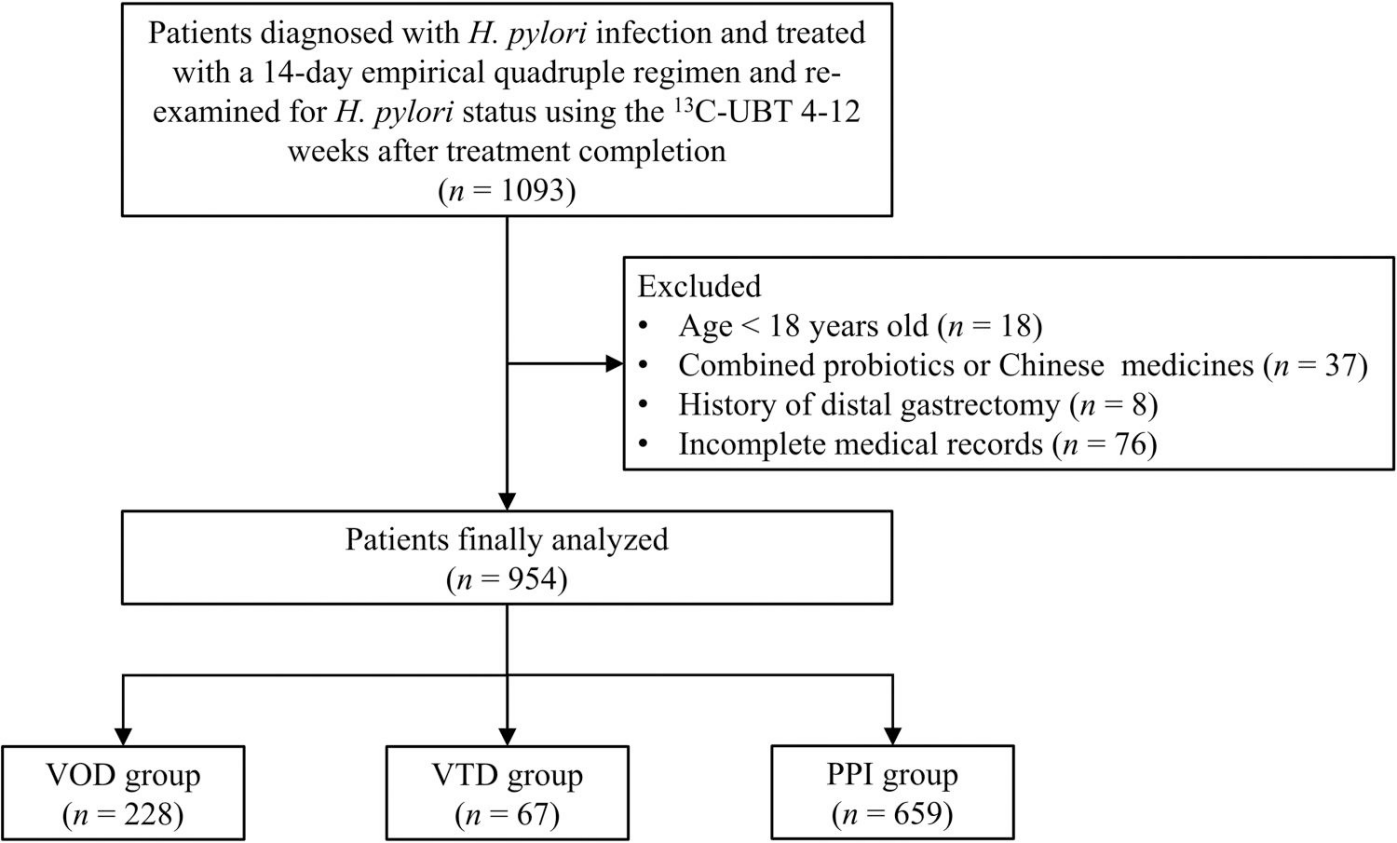
Flow chart of patient enrollment. Following the different acid suppressants contained in the quadruple regimen, patients included were classified into the low dosing frequency (20 mg daily) of vonoprazan (VOD group), high dosing frequency (20 mg twice daily) of vonoprazan (VTD group), and standard-dose PPI twice daily (PPI group). *H.pylori* = *Helicobacter pylori*; UBT = urea breath test; PPI = proton pump inhibitor.

A total of 954 patients (435 male and 519 female) with a mean age of 42.3 (range, 18.0–83.0) years old were finally included in our study (Figure 1). No significant differences were found in age, sex, residential address, *H.pylori* detection methods, clinical symptoms, or endoscopic findings between the VOD and the other two groups (all *P *>* *0.05, Table 1). The proportion of patients with diabetes was significantly lower in the VOD group than in the VTD group (6.1% *vs* 16.4%, *P *=* *0.008). The proportion of current smokers was significantly lower in the VOD group than in the PPI group (6.6% *vs* 12.7%, *P *=* *0.011). Regarding eradication attempts, 888 (93.1%) patients received initial treatment and the remaining 66 (6.9%) patients received rescue therapy. Patients receiving rescue therapy in the VOD group were fewer than those in the VTD group (7.9% *vs* 29.9%, *P *<* *0.001) and more than those in the PPI group (7.9% *vs* 4.2%, *P *=* *0.032). A total of 331 patients (34.7%) underwent endoscopic evaluation before commencing eradication therapy. Fewer patients in the VOD group underwent endoscopy than those in the VTD group (35.5% *vs* 50.7%, *P *=* *0.025).

**Table 1.** Baseline characteristics of included patients (*n *=* *954)

Characteristics	VOD group (*n *=* *228)	VTD group (*n *=* *67)	PPI group (*n *=* *659)
Age, years, mean ± SD	42.2 ± 14.6	42.1 ± 14.6	42.4 ± 14.2
Age, years, *n* (%)			
≤60	197 (86.4)	60 (89.6)	565 (85.7)
>60	31 (13.6)	7 (10.4)	94 (14.3)
Male, *n* (%)	97 (42.5)	32 (47.8)	306 (46.4)
Residential address, *n* (%)			
Urban area	174 (76.3)	50 (74.6)	528 (80.1)
Suburban area	54 (23.7)	17 (25.4)	131 (19.9)
Diabetes, *n* (%)	14 (6.1)	11 (16.4)	33 (5.0)
Current smoking, *n* (%)	15 (6.6)	1 (1.5)	84 (12.7)
Clinical symptoms, *n* (%)			
Abdominal pain/distension	76 (33.3)	29 (43.3)	234 (35.5)
Nausea/bitter taste in the mouth	7 (3.1)	2 (3.0)	31 (4.7)
Acid reflux and heartburn	8 (3.5)	4 (6.0)	22 (3.3)
Physical examination	119 (52.2)	26 (38.8)	331 (50.2)
Other	18 (7.9)	6 (9.0)	41 (6.2)
Detection method, *n* (%)			
^13^C- UBT	170 (74.6)	51 (76.1)	511 (77.5)
RUT	58 (25.4)	16 (23.9)	148 (22.5)
Eradication treatment attempt, *n* (%)			
Initial	210 (92.1)	47 (70.1)	631 (95.8)
Rescue	18 (7.9)	20 (29.9)	28 (4.2)
Received gastroscopy before eradication, *n* (%)	81 (35.5)	34 (50.7)	216 (32.8)
Endoscopic and pathological findings^a^, *n* (%)			
Atrophic gastritis (closed type)	21 (25.9)	5 (14.7)	51 (23.6)
Atrophic gastritis (open type)	2 (2.5)	0 (0)	2 (0.9)
Pre-neoplastic lesions after ESD	1 (1.2)	0 (0)	3 (1.4)
Peptic ulcer	12 (14.8)	10 (29.4)	57 (26.4)
Gastritis	45 (55.6)	19 (55.9)	103 (47.7)

a A total of 331 patients received endoscopic evaluation.

VOD = the low dosing frequency (20 mg daily) of vonoprazan; VTD = the high dosing frequency (20 mg twice daily) of vonoprazan; PPI = proton pump inhibitor; SD = standard deviation; UBT = urea breath test; RUT = rapid urease test; ESD = endoscopic submucosal dissection.

### Total *Helicobacter pylori* eradication rates (primary outcome)

Eradication was successful in 875 (91.7%) patients. The total eradication rates in the VOD, VTD, and PPI groups were 92.1%, 94.0%, and 91.4%, respectively. There was no significant difference between the VOD and the other two groups (all *P *>* *0.05, Table 2). In addition, multivariate-adjusted models were used to validate our findings. Model 1 was adjusted for age and sex, whereas Model 2 included additional adjustments for residence, smoking status, diabetes, and eradication attempts. The multivariate-adjusted analyses confirmed that the total eradication efficacy of the VOD group was comparable to those of the other two groups (all *P *>* *0.05; Table 3).

**Table 2.** *Helicobacter pylori* eradication rates of different groups

Rate	VOD group (*n *=* *228)	VTD group (*n *=* *67)	*P*1 value	PPI group (*n *=* *659)	*P*2 value
Total eradication rate, %	92.1 (210/228)	94.0 (63/67)	0.793^a^	91.4 (602/659)	0.724
Stratification of eradication attempts					
Initial eradication rate, %	93.3 (196/210)	95.7 (45/47)	0.744^a^	92.1 (581/631)	0.552
Rescue eradication rate, %	77.8 (14/18)	90.0 (18/20)	0.395^a^	75.0 (21/28)	1.000^a^
Stratification of age					
≤60 years old, %	93.9 (185/197)	95.0 (57/60)	1.000^a^	93.5 (528/565)	0.868
>60 years old, %	80.6 (25/31)	85.7 (6/7)	1.000^a^	78.7 (74/94)	0.819

a Fisher exact test.

VOD = the low dosing frequency (20 mg daily) of vonoprazan; VTD = the high dosing frequency (20 mg twice daily) of vonoprazan; PPI = proton pump inhibitor; *P*1 value = the VOD group vs VTD group; *P*2 value = the VOD group vs PPI group.

**Table 3.** The risk of *Helicobacter pylori* eradication failure and adverse events among different groups

Variable	Crude		Model 1^a^		Model 2^b^	
	OR (95% CI)	*P*	Adjust OR (95% CI)	*P*	Adjust OR (95% CI)	*P*
Total eradication failure						
VOD group	1 (reference)		1 (reference)		1 (reference)	
VTD group	0.741 (0.242–2.269)	0.741	0.761 (0.248–2.337)	0.634	0.739 (0.238–2.289)	0.599
PPI group	1.105 (0.636–1.920)	0.724	1.106 (0.635–1.924)	0.723	1.094 (0.626–1.912)	0.752
Initial eradication failure						
VOD group	1 (reference)		1 (reference)		1 (reference)	
VTD group	0.638 (0.150–2.714)	0.744	0.636 (0.150–2.696)	0.540	0.492 (0.184–1.314)	0.157
PPI group	1.189 (0.671–2.105)	0.653	1.179 (0.667–2.083)	0.570	1.253 (0.763–2.060)	0.373
Rescue eradication failure						
VOD group	1 (reference)		1 (reference)		1 (reference)	
VTD group	0.450 (0.009–2.170)	0.395	0.457 (0.094–2.215)	0.331	0.458 (0.109–1.923)	0.276
PPI group	1.125 (0.383–3.300)	1.000	1.152 (0.384–3.451)	0.801	0.919 (0.326–2.596)	0.874
Adverse event rate						
VOD group	1 (reference)		1 (reference)		1 (reference)	
VTD group	2.024 (1.032–3.968)	0.038	2.072 (1.053–4.077)	0.035	2.073 (1.054–4.078)	0.035
PPI group	0.689 (0.458–1.035)	0.072	0.708 (0.470–1.066)	0.098	0.703 (0.467–1.058)	0.091

a Model 1 was adjusted for sex and age (≤ 60 or > 60 years old).

b Model 2 was adjusted for model 1 plus eradication attempts, residential address, current smoking status, and diabetes when they were not the strata variables.

VOD = the low dosing frequency (20 mg daily) of vonoprazan; VTD = the high dosing frequency (20 mg twice daily) of vonoprazan; PPI = proton pump inhibitor; OR = odds ratio; CI = confidence interval.

### Eradication rates in subgroup analyses (secondary outcome)

#### Stratification by eradication attempts

In our cohort, the eradication attempts showed a significant difference among the three groups (*P *<* *0.001). Therefore, further subgroup analyses stratified by the number of eradication attempts were performed. The initial eradication rates in the VOD, VTD and PPI groups were 93.3%, 95.7%, and 92.1%, respectively. No significant differences were found between the VOD group and the other two groups (both *P *>* *0.05, Table 2). Regarding rescue therapy, the VOD group also had a rescue eradication rate comparable to that of the PPI group (77.8% *vs* 75.0%, *P *=* *1.000). Although the VOD group had a lower rescue eradication rate than the VTD group, the difference was not statistically significant (77.8% *vs* 90.0%, *P *=* *0.395). Multivariate analyses (both Models 1 and 2) further confirmed that for both initial and rescue treatments, patients treated with the VPZ (20 mg daily)-based quadruple regimen did not experience a significantly increased risk of eradication failure compared with the corresponding VPZ (20 mg twice daily) or PPI (standard dose twice daily)-based regimens (all *P *>* *0.05, Table 3).

#### Stratification by acid suppressors

**Figure 2.**
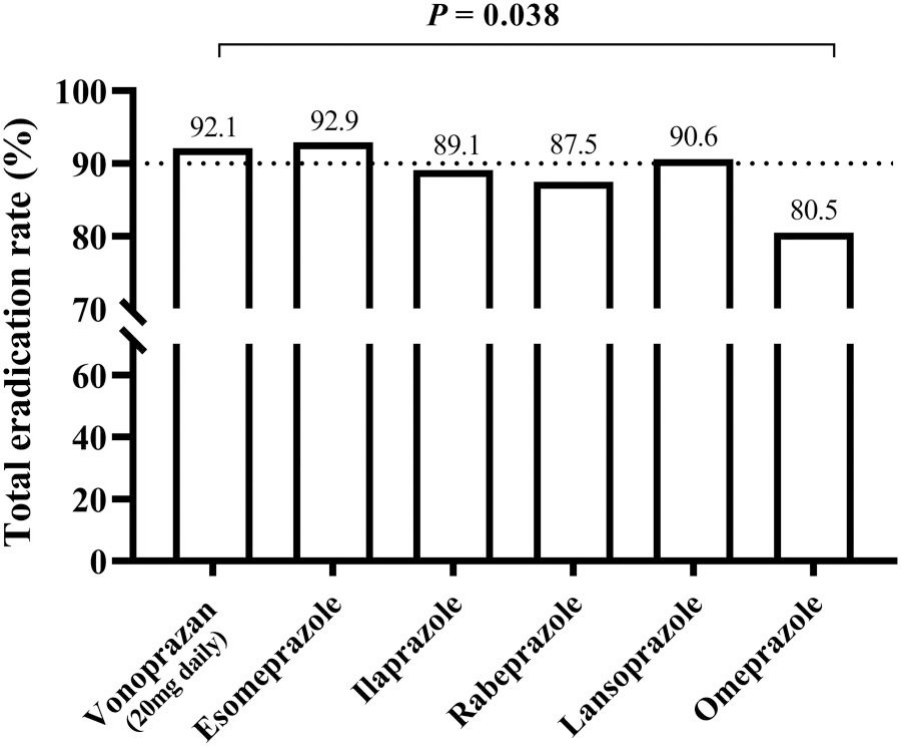
*Helicobacter pylori* eradication rates stratified by acid suppressors. The eradication rate of a low dosing frequency of vonoprazan-containing regimen was comparable to those of esomeprazole-, ilaprazole-, rabeprazole-, or lansoprazole-containing regimen, and significantly higher than that in the omeprazole-containing regimen.

Five different first- and new-generation PPIs were included in the study (Figure 1). To further compare the eradication efficacy of VPZ (20 mg daily) with those of the five different PPIs, a subgroup analysis stratified by acid suppressors was performed. As shown in Supplementary Table 1, VPZ (20 mg daily) showed eradication efficacy comparable to that of esomeprazole, ilaprazole, rabeprazole, and lansoprazole (all *P *>* *0.05). VPZ (20 mg daily) achieved a significantly higher eradication rate than omeprazole (92.1% *vs* 80.5%, *P *=* *0.038; Figure 2).

#### Stratification by age

Age is a potential risk factor for *H.pylori* eradication rates due to *H.pylori* antibiotic resistance. Therefore, a subgroup analysis stratified by age was performed to compare the eradication efficacy among the VOD, VTD, and PPI groups. As shown in Table 2, eradication rates showed a decreasing trend in patients aged > 60 years; however, the eradication efficacy showed no significant difference between the VOD and VTD groups or between the VOD and PPI groups in the various age subgroups (all *P *>* *0.05).

### Risk factors for eradication failure (secondary outcome)

Logistic regression analysis was performed to explore potential risk factors for eradication failure. Univariate analysis revealed that age > 60 years and use of rescue therapy were significantly associated with eradication failure. Further multivariate analysis confirmed that age > 60 years (OR = 2.165, 95% CI: 1.188–3.945, *P *=* *0.012, Table 4) and rescue therapy (OR = 3.496, 95% CI: 1.784–6.848, *P *<* *0.001) were independent risk factors for eradication failure, whereas VPZ at a low dosing frequency of 20 mg daily in the quadruple regimen was not (*P *=* *0.779).

**Table 4.** Risk factors for *Helicobacter pylori* eradication failure

Variable	Univariate analysis		Multivariate analysis	
	OR (95% CI)	*P*	OR (95% CI)	*P*
Age > 60 years old (yes vs no)	1.927 (1.087–3.417)	0.023	2.165 (1.188–3.945)	0.012
Gender (female vs male)	1.120 (0.703–1.782)	0.633		
Residential address (suburban area vs urban area)	0.579 (0.300–1.117)	0.100		
Diabetes (yes vs no)	1.301 (0.540–3.131)	0.469	1.165 (0.463–2.931)	0.745
Current smoking (yes vs no)	0.818 (0.365–1.829)	0.623	0.932 (0.408–2.129)	0.867
Clinical symptoms (yes vs no)	1.277 (0.804–2.030)	0.299		
Acid suppressants of vonoprazan 20 mg daily (yes vs no)	0.934 (0.540–1.617)	0.808	0.919 (0.509–1.659)	0.779
Eradication attempts (rescue vs initial)	2.981 (1.546–5.747)	0.001	3.496 (1.784–6.848)	<0.001

OR = odds ratio; CI = confidence interval.

### Adverse events (secondary outcome)

A total of 787 patients (82.5%) were followed up in our study (mean follow-up time, 6.7 ± 2.0 months), with 161 (70.6%) from the VOD group, 48 (71.6%) from the VTD group, and 578 (87.7%) from the PPI group. The mean follow-up period was 6.7 ± 2.0 months. The AE rate in the VOD group was significantly lower than that in the VTD group (26.1% *vs* 41.7%, *P *=* *0.038; Table 5) but not significantly different from that in the PPI group (26.1% *vs* 19.6%, *P *=* *0.072). The gastrointestinal AEs associated with dyspepsia were less frequent in the VOD group than in the VTD group (3.7% *vs* 16.7%, *P *=* *0.004). Crude analysis, as well as multivariate analyses, further confirmed that patients in the VOD group experienced significantly lower AE rates than those in the VTD group (all *P *<* *0.05, Table 3). In the adjusted Model 2, the OR of patients developing AEs was 2.073 (95% CI, 1.054–4.078, *P *=* *0.035, Table 3) in the VTD group compared with the VOD group.

**Table 5.** Adverse events of different groups

Variable	VOD group (*n *=* *161)	VTD group (*n *=* *48)	*P*1 value	PPI group (*n *=* *578)	*P*2 value
Patients with AEs, *n* (%)	42 (26.1)	20 (41.7)	0.038	113 (19.6)	0.072
Gastrointestinal AEs, *n* (%)					
Abdominal pain	7 (4.3)	4 (8.3)	0.280^a^	18 (3.1)	0.444
Abdominal distention	4 (2.5)	1 (2.1)	1.000^a^	10 (1.7)	0.518^a^
Constipation	6 (3.7)	1 (2.1)	1.000^a^	11 (1.9)	0.229^a^
Diarrhea/soft stool	8 (5.0)	6 (12.5)	0.095^a^	15 (2.6)	0.125
Dyspepsia	6 (3.7)	8 (16.7)	0.004^a^	13 (2.2)	0.272^a^
Reflux/heartburn	9 (5.6)	5 (10.4)	0.320^a^	22 (3.8)	0.318
Nausea/vomiting	3 (1.9)	1 (2.1)	1.000^a^	11 (1.9)	1.000^a^
Extra-gastrointestinal AEs, *n* (%)					
Skin rash	4 (2.5)	0 (0.0)	0.576	9 (1.6)	0.495
Taste disturbance	10 (6.2)	4 (8.3)	0.742^a^	19 (3.3)	0.091
Headache/dizziness	2 (1.2)	1 (2.1)	0.545^a^	8 (1.4)	1.000^a^
Anorexia	1 (0.6)	0 (0.0)	1.000^a^	5 (0.9)	1.000^a^
Others	1 (0.6)	2 (4.2)	0.133^a^	10 (1.7)	0.472^a^

a Fisher exact test.

VOD = the low dosing frequency (20 mg daily) of vonoprazan; VTD = the high dosing frequency (20 mg twice daily) of vonoprazan; PPI = proton pump inhibitor; AEs = adverse events; *P*1 value = the VOD group vs VTD group; *P*2 value = the VOD group vs PPI group.

## Discussion

Our findings suggest that VPZ at a low dosing frequency of 20 mg daily is a comparable alternative to VPZ administered at 20 mg twice daily and PPIs administered at standard dose twice daily in empirical quadruple regimens. The VOD group yielded eradication rates comparable to those of the VTD and PPI groups (all *P *>* *0.05). Age > 60 years and use of rescue therapy were independent risk factors for eradication failure, whereas VPZ at a low dosing frequency of 20 mg daily in the quadruple regimen was not. To our knowledge, this is the first large-cohort retrospective study to evaluate the effectiveness of low-dose VPZ in an empirical quadruple regimen.

The favorable eradication efficacy of VPZ in dual and triple regimens has been demonstrated in previous studies [15, 16]. In particular, the latest studies using Chinese population data also reported that VPZ and amoxicillin dual therapy are potential first-line treatments for *H.pylori* infection [17, 18]; however, our findings demonstrated that VPZ is also a valuable component of the quadruple regimen. In our study, VPZ (20 mg daily)-based empirical quadruple regimens achieved a satisfactory initial eradication rate of 93.3% and a rescue eradication rate comparable to that of PPIs (77.8% *vs* 75.0%, *P *=* *1.000). The rescue eradication rate of the PPI-containing quadruple regimen in our study was consistent with the 75.0%–85.1% reported in previous studies [19, 20]. Even in the subgroup analysis stratified by PPIs, VPZ (20 mg daily) was a favorable alternative to the standard twice-daily dose of new-generation PPIs (esomeprazole, ilaprazole, and rabeprazole) (all *P *>* *0.05) and was significantly superior to omeprazole (92.1% *vs* 80.5%, *P *=* *0.038). Our findings are partially consistent with those of a recent study by Lu *et al.* [21], who suggested that VPZ (20 mg daily) had comparable initial eradication efficacy to esomeprazole (20 mg twice/day) in a quadruple regimen. The more profound and persistent acid suppression of VPZ compared with that of PPIs may largely explain our finding that a low dosing frequency of VPZ had comparable efficacy to a double dosing frequency of PPIs in the empirical quadruple regimen. Pharmacokinetic studies have reported that VPZ 20 mg achieved a more rapid and sustained acid inhibitory effect vs the standard dose of esomeprazole 20 mg or rabeprazole 10 mg and even achieved equivalent acid inhibition (mainly pH ≥ 5 holding time ratios) as compared with esomeprazole (20 mg twice daily) [22, 23].

Currently, there is no consensus regarding the optimal frequency of VPZ administration in quadruple therapy. Almost all previous studies have used a VPZ dose of 20 mg twice daily for the eradication of *H.pylori*. A previous meta-analysis suggested that high-dose PPIs were more effective than standard doses for *H.pylori* eradication [24], which suggests that a quadruple regimen based on VPZ (20 mg twice daily) may achieve higher eradication efficacy than a low frequency of VPZ (20 mg daily), especially for rescue eradication; however, the optimal dosing frequency of VPZ has not been explored before. A recent randomized clinical trial in patients with gastroesophageal reflux disease suggested that 20 and 40 mg of VOD possessed similar acid inhibition efficacy [25]. In our study, there was no evidence that a higher frequency of VPZ (20 mg twice daily) in the quadruple regimen significantly improved the initial or rescue eradication rates. Although the VPZ (20 mg twice daily)-based quadruple regimen showed a higher rate of rescue eradication than VPZ alone (20 mg daily), the difference was not statistically significant (90.0% *vs* 77.8%, *P *=* *0.395). A pharmacological study, based on 24-h gastric pH monitoring, found that VPZ caused sustained gastric acid inhibition throughout the 24-h period in both single and multiple repeat-dosing studies when dosed at ≥ 20 mg [23]. Maintenance of a gastric pH ≥ 5 is essential for eradication. The pH ≥ 5 holding time ratio of VPZ 20 mg daily has been reported to be as high as 91% [22]. These findings may largely explain our finding that VPZ at a low dosing frequency of 20 mg daily in quadruple therapy could provide adequate acid inhibition for *H.pylori* eradication, whereas a high dosing frequency of VPZ (20 mg twice daily) did not achieve significantly higher efficacy. Future prospective studies are warranted to validate these findings.

Several factors such as poor patient adherence, antibiotic resistance, inadequate acid inhibition, prior treatment history, and older age have been identified as predictors of potential eradication failure [8, 26]. Consistent with previous studies, we found that age > 60 years and rescue therapy were independent risk factors for eradication failure, whereas VPZ (20 mg/day) was not. This further suggests that VPZ at a low frequency is feasible and acceptable for empirical quadruple therapy.

In terms of AEs, 175 (22.2%) of the 787 patients reported mild AEs; however, no severe or fatal AEs occurred. Patients who received the VPZ (20 mg twice/day)-based quadruple regimen reported more AEs than those who received VPZ (20 mg daily) (OR = 2.073, 95% CI: 1.054–4.078, *P *=* *0.035), especially dyspepsia (3.7% *vs* 16.7%, *P *=* *0.004). VPZ (20 mg daily) is an adequate and recommended dose for the treatment of gastric acid-related diseases with favorable clinical effects, particularly in gastroesophageal reflux disease. It is not difficult to understand that potent and sustained inhibition of gastric acid secretion by a doubled frequency VPZ (20 mg twice daily) may lead to excessive inhibition of gastric acid secretion, resulting in dyspepsia associated with acid deficiency. Regarding extra-gastrointestinal AEs, taste disturbance was the most common adverse reaction induced by VPZ, with a rate of 6.2% in the VOD group and 8.3% in the VTD group. This is consistent with previous studies reporting a range of 4.0%–18.2% [13, 27].

Our study has several limitations. First, as a single-center retrospective study, our findings may have limited generalizability. However, as the first real-world study, we believe that our findings have a suggestive effect on clinical practice. Second, as a real-world retrospective study, it is a great pity that the data on antibiotic susceptibility were missing in our study because it is not recommended for routine diagnosis in the guidelines [28]. Third, the number of patients who underwent rescue therapy included in our study was relatively small. Moreover, it is undeniable that rescue therapy is strongly associated with poor patient compliance. Patients with poor compliance often fail to recheck *H.pylori* infection status on time, leading to missing rescue therapy data. A prospective study with a large sample size of patients receiving rescue therapy can overcome this limitation.

## Conclusions

VPZ administered at a low dose of 20 mg daily is an effective and safe component of quadruple therapy for *H.pylori* eradication. Older age and rescue therapy were independent risk factors for eradication failure, whereas low-dose VPZ (20 mg daily) was not.

## Supplementary Material

goae036_Supplementary_Data
