# Supplementary material for: The efficacy and safety of vonoprazan in quadruple therapy for Helicobacter pylori eradication: a comparative study
Source: Gastroenterol Rep (Oxf). 2024 Apr 15;12:goae036. doi: 10.1093/gastro/goae036 (PMC11018536; doi:10.1093/gastro/goae036)
Supplement: goae036_Supplementary_Data [file goae036_supplementary_data.docx]

**Supplementary Material**

**Supplement Table 1.** *Helicobacter pylori* eradication rates of quadruple therapy classified by acid suppressors

| Acid suppressors | Total eradication rate, % | *P* value^b^ |
| --- | --- | --- |
| Vonoprazan^a^ | 92.1 (210/228) | / |
| New generation PPI | |  |
| Esomeprazole | 92.9 (406/437) | 0.707 |
| Ilaprazole | 89.1 (41/46) | 0.558^†^ |
| Rabeprazole | 87.5 (7/8) | 0.494^†^ |
| First generation PPI | |  |
| Lansoprazole | 90.6 (115/127) | 0.614 |
| Omeprazole | 80.5 (33/41) | 0.038^†^ |

PPI, proton pump inhibitor.

^†^Fisher exact test.

^a^A low dose frequency of vonoprazan (20 mg daily).

^b^Vonoprazan vs PPI.
